# Nanozyme Based on Dispersion of Hemin by Graphene Quantum Dots for Colorimetric Detection of Glutathione

**DOI:** 10.3390/molecules27206779

**Published:** 2022-10-11

**Authors:** Zhaoshen Li, Xiaochun Deng, Xiaoping Hong, Shengfa Zhao

**Affiliations:** 1 Guangxi Medical University Cancer Hospital, Guangxi Medical University, Nanning 530021, China; 2 Key Laboratory of Surface & Interface Science of Polymer Materials of Zhejiang Province, Department of Chemistry, Zhejiang Sci-Tech University, Hangzhou 310018, China

**Keywords:** nanozyme, graphene quantum dot, hemin, peroxidase-like activity, colorimetric determination of glutathione

## Abstract

Compared with natural enzymes, nanozymes have the advantages of good catalytic performance, high stability, low cost, and can be used under extreme conditions. Preparation of highly active nanozymes through simple methods and their application in bioanalysis is highly desirable. In this work, a nanozyme based on dispersion of hemin by graphene quantum dot (GQD) is demonstrated, which enables colorimetric detection of glutathione (GSH). GQD was prepared by a one-step hydrothermal synthesis method. Hemin, the catalytic center of heme protein but with low solubility and easy aggregation that limits its catalytic activity, can be dispersed with GQD by simple sonication. The as-prepared Hemin/GQD nanocomplex had excellent peroxidase-like activity and can be applied as a nanozyme. In comparison with natural horseradish peroxidase (HRP), Hemin/GQD nanozyme exhibited a clearly reduced Michaelis–Menten constant (*K*_m_) when tetramethylbenzidine (TMB) was used as the substrate. With H_2_O_2_ being the substrate, Hemin/GQD nanozyme exhibited a higher maximum reaction rate (*V*_max_) than HRP. The mechanisms underlying the nanozyme activity were investigated through a free radical trapping experiment. A colorimetric platform capable of sensitive detection of GSH was developed as the proof-of-concept demonstration. The linear detection range was from 1 μM to 50 μM with a low limit of detection of 200 nM (S/N = 3). Determination of GSH in serum samples was also achieved.

## 1. Introduction

Natural enzymes are important biocatalysts because they are closely related to life processes through controlling many catalytic processes such as metabolism, nutrition and energy conversion. Due to the efficient and specific catalysis of enzymes, chemical reactions in living organisms can be carried out under very mild conditions. However, most enzymes are proteins that are prone to structural changes in non-physiological environments such as acid or alkaline conditions and heat, resulting in loss of catalytic activity [1]. In addition, natural enzymes are also easily digested and degraded by proteases in the environment. With the advancements of biochemistry, chemistry and materials chemistry, artificial enzymes with low cost, simple preparation, high efficiency and good stability have attracted great attention [2,3,4].

The nanozyme is a new generation of artificial enzyme [5]. Like natural enzymes, a nanozyme can efficiently catalyze the reaction of substrates, and has similar catalytic properties (e.g., substrate specificity, catalytic efficiency) and enzymatic properties (e.g., enzymatic reaction kinetics) [6,7,8]. In addition, a nanozyme is more stable than natural enzymes. Thus, it can still maintain high catalytic activity in more extreme environments (e.g., strong acid or alkali conditions, and higher temperature) [9,10,11]. As nanomaterials with enzymatic activity, nanozymes also have unique physicochemical properties of nanomaterials, such as magnetic and luminescence properties, providing convenience for the design of complex multifunctional catalytic systems [12,13]. Therefore, the nanozyme has great potential as a substitute for natural enzymes in biosensing, environmental monitoring, agricultural analysis, medical detection and other fields [8,9]. The synthesis of efficient nanozymes by facile and controllable methods is of great significance.

Hemin, an iron porphyrin compound (C_34_H_32_ClFeN_4_O_4_), is the prosthetic group of hemoglobin, related also to myoglobin, cytochrome, peroxidase, or catalase, etc. As a coordination chelate between iron atom and four pyrrole groups on the porphyrin ring, hemin possesses peroxidase-mimicking activity [14]. However, hemin has a very low solubility in water. Although it is soluble in strong alkaline solutions (e.g., sodium hydroxide), hemin tends to aggregate to form dimers in aqueous solutions, losing catalytic activity. Therefore, it is a great challenge to directly use hemin as a molecular catalyst [15,16]. To solve these problems, scientists tried to synthesize iron porphyrin derivatives to improve the water dispersibility. However, this strategy often suffers from complex synthesis and complicated purification. In contrast, some materials with high specific surface area can complex with hemin to improve its dispersion and stability in water [14,17]. It has been reported that 2D carbon materials can make complexes with hemin through non-covalent interactions including π-π conjugation or electrostatic interactions, leading to enhanced activity and stability. For example, hemin can be composited with porous graphitic carbon nitride (g-C_3_N_4_) nanosheets or graphene, and the resulting nanomaterials have good water dispersibility and can be used as peroxidase-mimicking for colorimetric determination of glucose, mononuclear glycosides or as highly active oxidation catalysts [14,17,18]. Although these methods solve the problem of the dispersibility of hemin in water, the material preparation process is relatively complicated and involves some toxic raw materials. Therefore, development of simple, environmentally friendly and low-cost nanomaterials to improve the dispersion/stability in water, and the peroxidase-mimicking activity of hemin is highly desirable. 

Graphene based nanomaterials have attracted much attention due to their unique structure, multidimensional scale (e.g., 0D quantum dots [19,20,21], 1D nanoribbons [22] and nanotubes [23], 2D nanosheets [24], 3D foam [25,26,27]) and excellent physicochemical properties. Until now, these nanomaterials have been widely used as functional elements for the development of high-performance chemo/biosensors [28,29,30,31,32]. As the latest addition to the graphene nanomaterial family, the graphene quantum dot (GQD) is a zero-dimensional graphene material with an ultra-small size below 10 nm and single layer or a few layers of graphene sheets [33]. Due to its unique structural and optical properties (photoluminescence and electroluminescence), the GQD has shown broad application potential in the fields of chemical/biological sensing [34,35,36], and catalysis [37,38,39,40], etc. In addition, the ultra-small size of GQD leads to a high proportion of edge groups. Therefore, the GQD usually has excellent water solubility because of the hydrophilic groups (e.g., oxygen-containing groups) at the edges of GQD [41]. In addition, GQDs can also interact with functional small molecules through non-covalent interactions (e.g., electrostatic interactions and π-π interactions) to prepare functional composites [42,43]. Combined with the excellent biocompatibility and high stability of carbon materials, GQD is expected to serve as an excellent matrix nanomaterial to facilitate the dispersion of functional molecules in water. 

In this work, graphene quantum dots are utilized to disperse hemin and construct a highly active nanozyme that can achieve colorimetric detection of glutathione (GSH). GQDs are easily prepared by a one-step hydrothermal synthesis with simple preparation method, high yield and low cost. Through simple sonication, the dispersion of hemin by GQDs can be achieved through non-covalent interactions. The as-prepared Hemin/GQD nanocomposite possesses excellent peroxidase-like activity and can be used as nanozyme. In comparison with natural horseradish peroxidase (HRP), Hemin/GQD nanozyme had a significantly reduced Michaelis constant (*K*_m_) when tetramethylbenzidine (TMB) was used as a substrate. In addition, a higher maximum reaction rate (*V*_max_) than HRP was also obtained with H_2_O_2_ being the substrate. The reactive oxygen groups in the enzymatic process were investigated to reveal the mechanism of nanozyme activity. As a proof-of-concept demonstration, a platform capable of colorimetric detection of glutathione (GSH) was developed, which can realize sensitive detection of GSH in serum.

## 2. Results and Discussion

### 2.1. Synthesis and Characterization of GQD and Hemin/GQD

**Figure 1:**
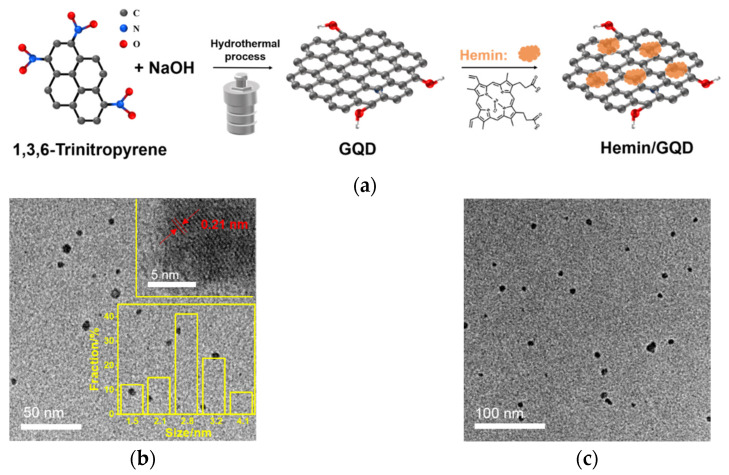
(**a**) Schematic illustration for the easy preparation of Hemin/GQD. (**b**) TEM image of GQD. The top inset is HRTEM image with indicated lattice parameter. The bottom inset is the size distribution. (**c**) TEM image of Hemin/GQD.

To achieve the dispersion of hemin, graphene quantum dots (GQDs) must be easily synthesized and possess excellent water dispersibility. Commonly, GQDs can be synthesized through “top-down” or “bottom-up” methods. Among them, the “top-down” strategy is to use chemical, electrochemical or physical methods to cut large, graphitized carbon materials (e.g., graphene, carbon nanotubes or carbon black, etc.) [44,45]. The latter is achieved by fusion or pyrolysis/carbonization of organic small molecules or precursors. Compared with “top-down” synthesis strategy, the “bottom-up” synthesis of GQD tends to exhibit higher yields, better uniformity, more easily tunable structures and properties, and lower costs [46,47,48]. Figure 1a is the schematic illustration of the synthesis of GQDs by a one-step hydrothermal method using a bottom-up strategy. Trinitropyrene, that has a graphene core, is used as the carbon precursor to synthesize GQDs in sodium hydroxide medium. After a hydrothermal reaction, dialysis purification, and freeze-drying, a reddish-brown GQD powder was obtained with a yield of 83.0%. The as-prepared GQD material exhibited excellent dispersibility in water. Even when the solution concentration was 5 mg/mL, the solution was stable for several months without precipitation. Since the GQD has an sp^2^ carbon skeleton structure, it can interact with hemin through non-covalent interaction such as hydrophobic interaction. So, Hemin/GQD nanocomposite can be easily obtained by simple sonication.

The size and dispersion of GQD and Hemin/GQD were characterized by TEM. As shown by transmission electron microscopy (TEM, Figure 1b), GQDs are uniform in size (~2.8 nm on average) with a lattice spacing of 0.21 nm, corresponding to the lattice spacing of the graphene (100) plane. In addition, no agglomeration of GQDs is observed. When the GQD is composited with hemin, its size remains basically unchanged (Figure 1c). No agglomeration of Hemin/GQD is observed. 

**Figure 2:**
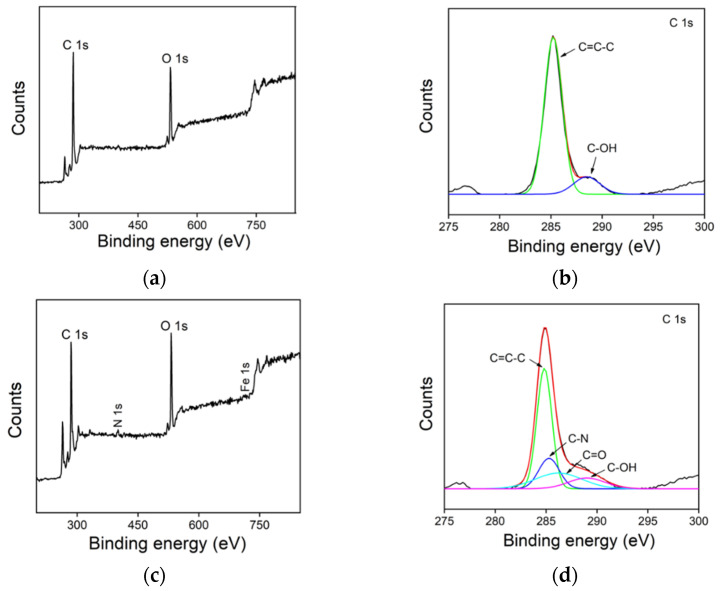
XPS survey spectrum (**a**) and high-resolution C1s spectrum (**b**) of GQD. XPS survey spectrum (**c**) and high-resolution C1s (**d**) spectrum of Hemin/GQD.

The composition of GQD and Hemin/GQD materials was characterized by X-ray photoelectron spectroscopy (XPS). The survey XPS spectrum of GQD shows two distinct peaks of C 1s and O 1s (Figure 2a), confirming the presence of C and O atoms. In the high-resolution spectrum of C1s (Figure 2b), the binding energy peak at 284.9 eV confirms the graphitic structure (C-C=C) and the peak at 288.3 eV could be assigned to sp^3^ C in C-OH groups. In the case of Hemin/GQD, very low characteristic signals of N and Fe elements also appear besides C and O in the survey XPS spectrum of Hemin/GQD (Figure 2c). To evaluate the N content, elemental (C, H, and N) analysis of Hemin/GQD was performed. The percentages of N and C are 4.8% and 36.3%, respectively. The low measured N signal in the XPS investigation might be ascribed to possible oxygen adsorption on the surface of Hemin/GQD, which could reduce the content of other elements. A low content of Fe is also observed on graphene quantum dots synthesized using hemin as the precursor [5]. In the high-resolution spectrum of N1s (Figure 2d), the C-N signal at 285.2 eV and C=O signal at 286.5 eV are attributed to the pyrrole nitrogen and carboxyl group of hemin.

**Figure 3:**
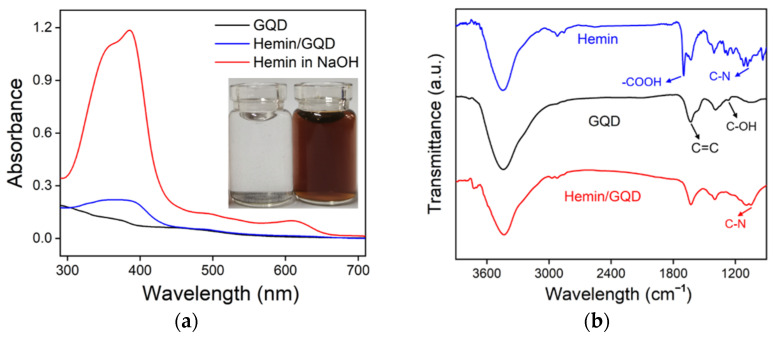
(**a**) UV absorption spectra of GQD (10 μg/mL), hemin (0.2 mM) in NaOH, and Hemin/GQD (10 μg/mL). Insets are the digital images of hemin in water (**left**) or Hemin/GQD (**right**). (**b**) FT-IR spectra of GQD, hemin and Hemin/GQD.

Hemin has a very low solubility in water. As shown in the inset in Figure 3a (left image), hemin settles to the bottom when it is added to water. The obtained solution is colorless. As hemin can be dissolved in strong alkaline solution, NaOH was used to dissolve it. A significant UV absorption peak is observed due to the existence of the pyrrole ring. When hemin is composited with GQDs, it can be completely dispersed in water. A dark brown solution is obtained (left image of inset in Figure 3a). In addition, the characteristic peak of hemin also appears in the UV absorption spectrum of Hemin/GQD, demonstrating the efficient composite of hemin on the GQDs. Fourier transform infrared spectroscopy (FT-IR) also verifies the efficient composite between hemin and GQDs. As shown in Figure 3b, the absorption at 1077 cm^−1^ corresponds to the C-N bond in hemin. In case of GQD, the absorption at 1270 cm^−1^ is assigned to the C-OH bond. These characteristic peaks appear in the spectrum of Hemin/GQD, proving the effective formation of the nanocomposite.

### 2.2. Peroxidase-Mimicking Activity of Hemin/GQDs

**Figure 4:**
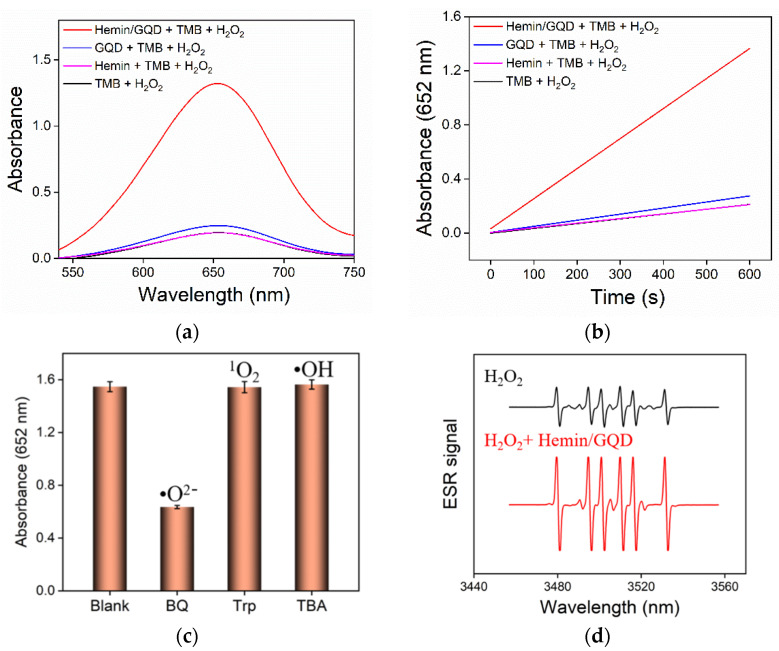
(**a**) Absorbance spectra of different mixture solutions after 10 min reaction. (**b**) Time-dependent change of absorbance at 652 nm of different mixture solutions. Curves obtained in TMB + H_2_O_2_ and Hemin + TMB + H_2_O_2_ in a and b are almost overlapping. (**c**) Absorbance values of the Hemin/GQD-TMB-H_2_O_2_ system in absence (blank) or presence of different ROS traps including 1,4-benzoquinone (BQ, 100 μM), tryptophan (Trp, 100 μg/mL) or tertbutanol (TBA, 100 μg/mL). (**d**) ESR spectra of H_2_O_2_ or H_2_O_2_ + Hemin/GQD in presence of DMPO.

As illustrated in Figure 4a, the peroxidase activity of Hemin/GQDs is confirmed by the catalyzed oxidization of tetramethylbenzidine (TMB) by H_2_O_2_, which produce oxidative TMB (oxTMB) with blue color and a characteristic peak at 652 nm. It is noteworthy that when hemin is added or not added to the blank solution of TMB + H_2_O_2_, the absorption spectrum or the change curve of A_652_ with reaction time almost overlap. This is due to the low solubility of hemin. In comparison with the weak reaction in the synthesis containing H_2_O_2_ and TMB, the addition of GQDs only results in low absorbance, indicating low peroxidase-mimicking activity. On the contrary, the system containing TMB, H_2_O_2_ and Hemin/GQD showed significantly high absorbance, demonstrating the intrinsic peroxidase-like activity of Hemin/GQD. By monitoring the change of absorbance at 652 nm with a UV-vis spectrometer, this catalytic reaction could be detected in a time-dependent manner (Figure 4b). Hemin/GQD shows a very high growth rate of absorbance. 

To understand the catalytic mechanism, the catalytic intermediates were studied (Figure 4c,d). The capture of reactive oxygen species (ROS) was performed. As shown in Figure 4c, three different radical scavengers were added to the Hemin/GQD + TMB + H_2_O_2_ system. Briefly, tertbutanol (TBA), tryptophan (Trp) and 1,4-benzoquinone (BQ) were applied as the indicators for hydroxyl radical (•OH), singlet oxygen (^1^O_2_) and superoxide anion free radicals (•O^2−^), respectively. When one of the three different radical scavengers was added to the Hemin/GQD + TMB + H_2_O_2_ system, it was found that only the solution with the addition of BQ has a significant decrease in absorbance, which proved that the main ROS generated by Hemin/GQD catalyzed decomposition of H_2_O_2_ was •O^2−^. In addition, DMPO was selected as the capture agent to perform electron paramagnetic resonance (EPR) measurement. As shown in Figure 4d, a significantly increased DMPO- •O^2−^ adduct signal was observed with a characteristic relative intensity of the peak (1:1:1:1:1:1) in presence of hemin. This further confirmed that Hemin/GQD mainly generated strong oxidizing •O^2−^ during the catalytic process.

### 2.3. Steady-State Kinetics of Hemin/GQD Nanozyme

**Figure 5:**
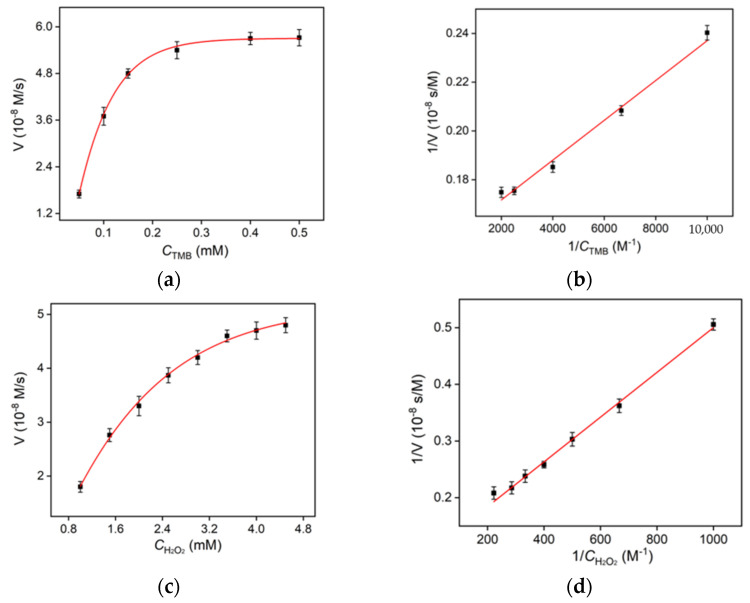
(**a**,**c**) Steady-state kinetic assay of GQDs using TMB (**a**) or H_2_O_2_ (**c**) as the substrate. The reaction velocity was determined through oxidation of TMB based on absorption at 652 nm with varying concentrations of (**a**) TMB or (**c**) H_2_O_2_. (**b**,**d**) Double-reciprocal plots of GQD activity obtained using Michaelis–Menten model at a fixed concentration of TMB H_2_O_2_ (**b**) or TMB (**d**) and different concentrations of TMB (**b**) or H_2_O_2_ (**d**).

The Michaelis-Menten model is employed to analyze the kinetic parameters of the Hemin/GQD nanozyme. As shown in Figure 5, the Michaelis-Menten constant (*K*_m_) and the maximum initial velocity (*V*_max_) were obtained from Lineweaver-Burk plot. As is well known, *K*_m_ is a constant which reflects the binding affinity between enzymes and substrates, and the *V*_max_ value reveals the turnover number of enzymes. Figure 5a,b show the steady-state kinetic curve and double-reciprocal curve of the Hemin/GQD catalytic reaction when TMB is used as the substrate. By using TMB as substrate, the *K*_m_ value and *V*_max_ value of hemin-GQD nanozyme are 4.623 × 10^−2^ mM and 6.369 × 10^−8^ M/s, respectively. In the case of native HRP [49], the *K*_m_ is 0.434 mM and the *V*_max_ is 10 × 10^−8^ M/s when TMB is used as the substrate. It was found that Hemin/GQD has a smaller *K*_m_ value, indicating that Hemin/GQD has a better adsorption capacity for TMB. By using H_2_O_2_ as substrate, the *K*_m_ value and *V*_max_ value of Hemin/GQD nanozyme are 4.54 mM and 10.61 × 10^−8^ M/s, respectively (Figure 5c,d). Compared with native HRP [50], the *K*_m_ value of Hemin/GQD is larger, while the *V*_max_ value increases nearly three-fold. Compared with other nanomaterials, Hemin/GQD has high peroxidase-mimicking activity. In comparison with other nanozymes based on carbon nanomaterials, our Hemin/GQD has the unique advantages of easy synthesis, and high catalysis performance.

### 2.4. Colorimetric Detection of GSH

Glutathione (GSH) is a kind of thiol tripeptide in cells, which exists widely in animals, plants, prokaryotic cells and other organisms. GSH is the most abundant cellular biothiol and the essential endogenous antioxidant in organisms, playing a central role in cellular defense against toxins and free radicals [51,52]. As is well known, an abnormal GSH level is closely related to cancer, liver injury, cardiovascular diseases and other diseases. For example, myocardial infarction, a common heart disease, is caused by continuous myocardial ischemia and hypoxia, which can trigger production of a large number of free radicals and then lead to the depletion of glutathione [53]. Therefore, highly sensitive and selective detection of GSH in serum is of great significance for the early diagnosis of acute myocardial infarction and other diseases.

**Figure 6:**
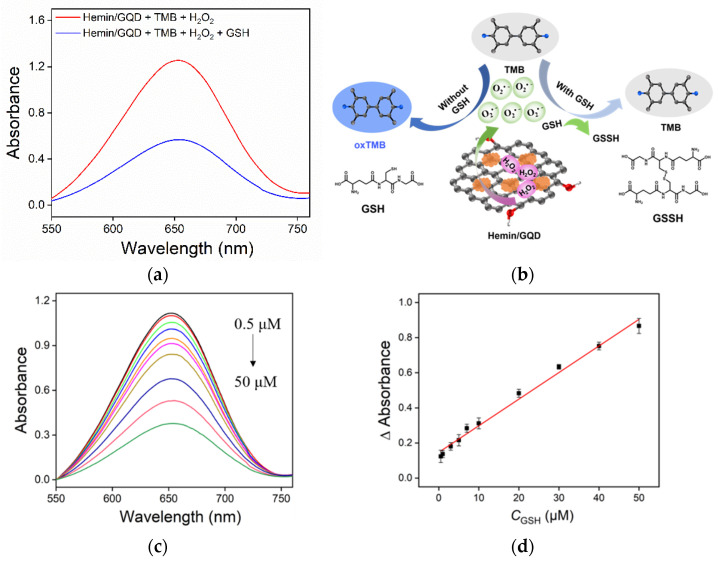
(**a**) Absorbance spectra obtained from the mixture of Hemin/GQD + TMB + H_2_O_2_ in the absence or presence of GSH (50 μM). (**b**) Schematic illustration for the colorimetric detection of GSH. (**c**) Absorbance spectra obtained from the mixture of GQD + TMB + H_2_O_2_ in the presence of different concentrations of GSH. (**d**) The linear calibration plot for colorimetric detection of GSH.

Based on the peroxidase-mimicking activity of Hemin/GQD, a colorimetric sensing for GSH is proposed. As shown in Figure 6a, the absorbance value of the solution significantly decreases after adding GSH in the TMB + H_2_O_2_ + Hemin/GQD solution. The mechanism of this phenomenon is illustrated in Figure 6b. As a peroxidase-minic, Hemin/GQD can catalyze the decomposition of hydrogen peroxide to generate reactive oxygen radicals •O^2−^, which further oxidizes TMB to generate blue oxTMB and generates characteristic absorption. In the presence of GSH, GSH competes with TMB for the strong oxidizing •O^2−^, resulting in a decrease in the content of oxTMB and a decrease in the absorbance at 652 nm.

As revealed in Figure 6c, the absorption value of oxTMB in the solution with Hemin/GQD catalyzed oxidation of TMB gradually decreases with the increase of GSH concentration. The decrease in absorbance at 652 nm (ΔAbsorbance) has a good linear relationship with the concentration of GSH (C_GSH_) in the range of 1–50 μM. The linear fitting equation is ΔAbsorbance = 0.0152C_GSH_(μM) + 0.147 (R^2^ = 0.990), where ΔAbsorbance is the absorbance difference of the solution before (A_0_) and after (A) adding GSH (Figure 6c). A limit of detection (LOD) of 0.194 μM is obtained using three signal-to-noise ratio (S/N = 3). Compared with other colorimetric detecting of GSH using other materials, this colorimetric detection based on Hemin/GQD has a lower LOD, indicating a sensitive detection of GSH. In addition, the low cost and simple synthesis of Hemin/GQD make this colorimetric method more advantageous.

### 2.5. Detection Selectivity and Real Sample Analysis 

**Figure 7:**
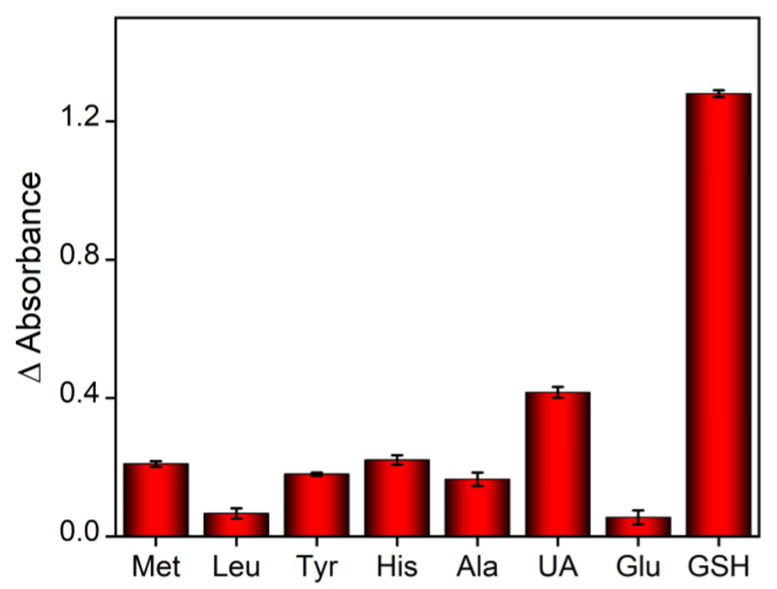
Change of absorbance after adding different substances to the Hemin/GQD + H_2_O_2_ + TMB system for 20 min. The concentrations for GSH and other substances are 20 μM and 100 μM, respectively.

The selectivity of the above colorimetric detection of GSH was further investigated. Biological samples usually contain common biological molecules. For instance, the basic component of serum is water, but serum also contains protein, fat, glucose, inorganic salts, amino acids/vitamins and other nutrients, as well as human metabolites (such as uric acid, the metabolite of purine). The colorimetric analysis based on the above peroxidase-minicking depends on whether the analyte can combine with the ROS generated in the catalytic process, inhibiting the oxidation of the substrate. Thus, proteins and fats that do not have reducibility will not interfere with the detection of GSH. The effect of other substances was examined. As shown in Figure 7, different kinds of possibly interfering substances including amino acids, uric acid, glucose and GSH were added to the TMB + H_2_O_2_ + Hemin/GQD system. To make the results clearer, the concentration of the above possible interfering substances (100 μM) is much higher than that of GSH (20 μM). In addition, a long reaction time (20 min) is employed. As shown, only the addition of GSH greatly changes the absorbance compared with the initial absorbance value of the solution, proving the selectivity of the detection. It is speculated that the above substances, even if they are glucose and uric acid with a certain reducibility, cannot combine with the ROS in the system, so they will not interfere with the detection. Determination of GSH in detal bovine serum was investigated using standard addition method. As shown in Table 1, the recovery ranged from 99.2% to 111% and the elative standard deviation (RSD) was no more than 3.3%.

## 3. Materials and Methods

### 3.1. Chemicals and Materials

Tetramethylbenzidine (TMB), 4-hydroxyethylpiperazineethanesulfonic acid (HEPES), hemin, d-methionine (Met), l-tyrosine Acid (Tyr), l-histidine (His), uric Acid (UA), glucose (Glu), glutathione (GSH), 1,4-benzoquinone (BQ), tryptophan (Trp), tertiary Butanol (TBA) were purchased from Shanghai Aladdin biochemical technology Co., Ltd. (Shanghai, China). Sodium hydroxide (NaOH) and methanol were purchased from Hangzhou Gaojing Fine Chemical Co., Ltd. (Hangzhou, China). l-leucine (Leu) and alanine (Ala) were purchased from Shanghai McLean Biochemical Technology Co., Ltd. (Shanghai, China). 5,5-dimethyl-1-pyrroline-*N*-oxide (DMPO) was obtained from Sigma-Aldrich Shanghai Trading Co., Ltd. (Shanghai, China). H_2_O_2_ was obtained from Tianjin Yongda Chemical Reagent Co., Ltd. (Tianjin, China).

### 3.2. Synthesis of GQD and Hemin/GQD 

Trinitropyrene was synthesized according to previous literature [54,55,56]. To synthesize GQDs, a mixture of trinitropyrene (2 mg/mL) and NaOH (0.4 M) was added to a Teflon reactor and reacted at 180 °C for 10 h. The obtained solution was firstly filtered with a 0.22 μm film to remove large-sized particles, and then fully dialyzed using a dialysis bag (with cut-off molecular weight of 500 Da) to remove unreacted small molecules. The dialysate was freeze-dried to obtain the GQD solid. Then, the GQD solid was dissolved in distilled water (1 mg/mL). Hemin (4 mM) was added to the GQD (10 μg/mL) solution and then ultrasonically dispersed for 1 h. After the obtained solution was centrifuged to remove possible solid particles, the resulting supernatant contained the Hemin/GQD.

### 3.3. Experiments and Instrumentations

Transmission electron microscopy (TEM) was conducted on a JEM-2100 transmission electron microscope (JEOL Ltd., Tokyo, Japan) at the operating voltage of 200 kV. The chemical composition of was performed by X-ray photoelectron spectroscopy (XPS) with PHI5300 electron spectrometer using 250 W, 14 kV, Mg Ká radiation (PE Ltd., Boston, MA, USA). UV-Vis absorption spectra were recorded on UV-2450 spectrophotometry (Shimadzu Corporation, Honshu Island, Japan). Electron paramagnetic resonance (EPR) measurements were recorded using an EMX-10/12 spectrometer (Bruker, Karlsruhe, Germany). 

### 3.4. Peroxidase-Mimicking Activity of Hemin/GQD

The nanozyme-catalyzed oxidation of H_2_O_2_ was monitored by UV-Vis spectrophotometer using TMB as the substrate. The used enzymatic reaction medium was HEPES buffer (0.1 M, pH = 4.0) containing TMB (0.3 mM) and H_2_O_2_ (4 mM). The peroxidase-mimicking activity of the nanozyme was determined by comparing the absorbance changes of the medium after adding GQD or Hemin/GQD. The concentration of GQD or Hemin/GQD was 3 μg/mL.

### 3.5. Determination of Steady-State Kinetic Constants of Nanozyme

The Michaelis constant *K*_m_ and the maximum reaction rate (*V*_max_) of Hemin/GQD, a peroxidase-mimicking product, were analyzed by using the double reciprocal curve of Michaelis equation. Briefly, the UV-Vis spectra of TMB + H_2_O_2_ + Hemin/GQD solution at 652 nm was recorded during different reaction times. In the experiment, the final concentration of Hemin/GQD was 3 μg/mL. TMB or H_2_O_2_ was used as the substrate, respectively. When TMB was used as the substrate, the concentration of H_2_O_2_ was set at 4 mM and the concentration of TMB ranged from 0.1 to 0.5 mM. When H_2_O_2_ was used as the substrate, the concentration of TMB was set at 0.3 mM and the concentration of H_2_O_2_ ranged from 1 to 5 mM.

### 3.6. Colorimetric Detection of GSH

For the colorimetric detection of GSH, HEPES buffer (0.1 M, pH = 4.0) containing TMB (0.3 mM), H_2_O_2_ (4 mM) and Hemin/GQD (3 μg/mL) was used as the detection medium. After adding different concentrations of GSH to the above reaction medium and reaction for 10 min, the absorbance at 652 nm was measured. For real sample analysis, GSH in fetal bovine serum was analyzed using standard addition method. Briefly, fetal bovine serum was diluted by a factor of 100 with HEPES buffer (0.1 M, pH = 4.0). Then, certain concentrations of GSH were added in the diluted serum samples. The obtained solution was introduced to the detection medium and reacted for 10 min before the absorbance at 652 nm was measured.

## 4. Conclusions

In summary, we developed a facile method for the preparation of peroxidase-mimicking nanozymes based on graphene quantum dot-dispersed hemin, which can achieve colorimetric detection of glutathione. GQDs were one-step synthesized using a bottom-up strategy. The preparation method is simple with an easily operated process and low cost. The Hemin/GQD nanocomposite can be achieved through the non-covalent interactions between GQDs and hemin. Hemin/GQD has excellent peroxidase-mimicking activity with a lower *K*_m_ (using TMB as substrate) and higher *V*_max_ (using H_2_O_2_ as substrate), and can be used to construct a colorimetric detection platform for sensitive detection of glutathione. This facile nanozyme synthesis strategy can greatly facilitate the practical application of enzyme mimics in the future. In addition, the fluorescent properties of GQD are also expected to provide more signal channel for the application of nanozymes. Based on the ultra-small size, good dispersibility, highly tunable surface and physicochemical properties and fluorescence properties of GQD, the application of GQD-based nanozymes is expected to be further expanded.

## Figures and Tables

**Table 1:** Determination of GSH in fetal bovine serum samples.

Sample ^a^	Added (μM)	Found (μM)	RSD (%)	Recovery (%)
Fetal bovine Serum ^a^	1.00	1.11	0.3	111
	5.00	4.96	2.6	99.2
	10.0	10.2	3.3	102

^a^ Samples with added GSH were diluted 100 times using HEPES (0.1 M, pH = 4). The concentration of GSH was the added concentration after dilution.

## Data Availability

The data presented in this study are available on request from the corresponding author.

## Sample Availability

Samples of the compounds are available from the authors.
